# 

**Published:** 1883-08

**Authors:** 


					﻿Whole Number,
CCCCLXIII.
August, 1883.
Number 2,
Vol. XLVII.
THE
C H IC AGO
MEDICAL
Journal & Examiner.
CHICAGO:
PUBLISHED BY THE CHICAGO MEDICAL PRESS ASSOCIATION
188 Clark Street.
ug'Read the Notice of this Journal among Advertisements.
				

